# Prevalence and factors associated with severe anaemia post-caesarean section at a tertiary Hospital in Southwestern Uganda

**DOI:** 10.1186/s12884-021-04157-x

**Published:** 2021-10-06

**Authors:** Sylvie Atosa Sivahikyako, Asiphas Owaraganise, Leevan Tibaijuka, David Collins Agaba, Musa Kayondo, Joseph Ngonzi, Julius Mugisha, Hamson Kanyesigye

**Affiliations:** 1 Department of Obstetrics and Gynaecology, Mbarara University of Science and Technology, P.O. Box 1410, Mbarara, Uganda; 2 Infectious Diseases Research Collaboration, Kampala, Uganda

**Keywords:** Severe anaemia, Day three, Post-caesarean section, Haemoglobin

## Abstract

**Background:**

Severe anaemia after caesarean section adversely affects the woman and the new-born. While prenatal anaemia is extensively studied, the literature on post-caesarean section anaemia is limited and characteristics of women at the highest risk of developing severe anaemia after caesarean section are unknown. This study aimed to determine the prevalence and factors associated with severe anaemia on day three post caesarean section.

**Methods:**

On the third day after caesarean section, women were consecutively enrolled in a cross-sectional study at Mbarara Regional Referral Hospital (MRRH). Women who got transfused peripartum were excluded. For every woman, we measured haemoglobin (Hb) concentration and collected data on sociodemographic, obstetric, and medical characteristics. The primary outcome was severe anaemia after caesarean section, defined as Hb < 7 g/dl. We used logistic regression analysis to determine factors associated with severe anaemia after caesarean section. *P*-value < 0.05 was considered statistically significant.

**Results:**

From December 2019 to March 2020, 427 of 431 screened women were enrolled in the study. Their mean age was 26.05 (SD ± 5.84) years. Three hundred thirteen (73.3%) had attended at least four antenatal care visits. The prevalence of severe anaemia post-caesarean section was 6.79%. Foetus with macrosomia (aOR 7.9 95%CI: 2.18–28.85, *p* <  0.01) and having mild or moderate anaemia pre-caesarean section (aOR:9.6, 95%CI: 3.91–23.77, *p* <  0.01) were the factors associated with severe anaemia after caesarean section.

**Conclusion:**

Severe anaemia in women post-caesarean section is relatively uncommon at our institution. It is associated with preoperative anaemia and macrosomic birth. Women with a low preoperative Hb concentration and those whose foetus have macrosomia could be targeted for haemoglobin optimisation before and during caesarean section.

**Supplementary Information:**

The online version contains supplementary material available at 10.1186/s12884-021-04157-x.

## Background

Anaemia is the decrease in the total count of the red blood cells (RBCs) or packed cell volume of RBCs or haemoglobin concentration below the reference values for the person’s age, sex, geographical location, and physiological status [1, 2], resulting into an impaired oxygen-carrying capacity of blood to the tissues. Anaemia remains a significant public health problem worldwide affecting 24.5–35.0% of women of reproductive age, especially in low-income countries (LICs) [3]. In Uganda, the prevalence of anaemia among women of childbearing age was 34% in 2016 [4]. In the state of pregnancy, physiological haemodilution reduces haemoglobin concentration during the first trimester, reaches a nadir in the second trimester before rising again in the third trimester [5]. The haemoglobin levels continue to rise during puerperium and peak in the immediate postpartum due to diuresis-induced resolution of pregnancy-induced anaemia plus redistribution of contracting uterine circulation to the systemic circulation [6]. Recovery to nonpregnant states occurs by 12 weeks post-delivery [7]. According to the World Health Organisation (WHO), pregnancy-specific haemoglobin levels are used to categorise anaemia in pregnancy; that is, 10–10.9 g/dl as mild anaemia, 7–9.9 g/dl as moderate, and < 7 g/dl as severe anaemia [8].

Maternal anaemia increases perinatal morbidity and mortality [9] including the risk of miscarriage, stillbirths, preterm birth, and low birth weight [10], the burden of depressive symptoms [11], and unfavourable mother-infant interactions [12]. Postpartum anaemia impairs wound healing, increases the risk for readmission and/or prolonged hospitalization, and increases the cost of care for families [13]. Peripartum anaemia is a crucial health issue due to increasing caesarean section rates in LICs [14].

The main risk factors for postpartum anaemia are pre-existing anaemia—especially due to iron deficiency combined with blood loss during delivery [15, 16]. Caesarean section has been shown to increase the risk of postpartum anaemia by twofold [17], due to the increased risk of uterine atony and severed vessels when opening the abdominal wall [18–20]. Incidental anaemia, especially in the third trimester, excessive intrapartum blood loss, younger women, and those not taking iron supplementation during puerperium have also been shown to predict postpartum anaemia [21].

Several studies on anaemia during pregnancy [22–24] have provided limited information on the prevalence of anaemia after delivery, especially, in women undergoing caesarean section in the context of increasing caesarean-section rates in LICs [14]. Given limited resources, most women are discharged after caesarean section with an unknown haemoglobin concentration. To skilfully identify women who are vulnerable to severe anaemia after caesarean section for appropriate intervention before hospital discharge, the prevalence of postpartum anaemia in each setting must be monitored and factors associated with anaemia after caesarean delivery evaluated. In addition, heterogeneity of peripartum population, knowledge and skills of medical personnel and logistical provision for caesarean section in different health facilities require new data on general trends of anaemia in women after delivery. This study aimed to determine the prevalence and factors associated with severe anaemia after caesarean section.

## Materials and methods

### Study design, site, and period

A cross-sectional study was conducted on the postnatal ward at Mbarara Regional Referral Hospital (MRRH) in Southwestern Uganda from December 2019 to March 2020. MRRH is a government-funded public tertiary hospital located along the Mbarara-Kabale road about 260 km south of Uganda’s capital Kampala. It serves as a referral centre for the estimated 5 m population of southwestern Uganda [25]. The MRRH records show approximately 9000 were delivered women in 2019—40% of which were caesarean sections. The caesarean section rate is high due to a big number of pregnant women who are referred from lower-level health facilities for operative delivery. The majority end up as emergency caesarean sections due to obstructed labour, malpresentation, repeat caesarean sections, multiple pregnancies, failed labour induction, placental anomalies and non-reassuring foetus. The hospital’s maternal mortality ratio stands at 261 per 100,000 live births [26]. Resident doctors in the Department of Obstetrics and Gynecology of Mbarara University of Science and Technology (MUST) conduct most of the caesarean deliveries. While Ugandan guidelines require a minimum of 24-h hospitalization postpartum [27], as a standard clinical practice at MMRH, post caesarean section, women are admitted to the postnatal ward until day three post-operative. We drew blood samples on the third day after delivery (72 h) to allow the minimum of 48 h to stabilize haemoglobin concentration following extra- and intravascular fluid volume changes during and shortly after delivery [16]. Postoperative anaemia is assessed clinically and laboratory testing offered at the discretion of the reviewing clinician.

### Study population

Women who underwent caesarean section at Mbarara Regional Referral Hospital were the target population of this study.

### Eligibility criteria

We included adult women and emancipated minors at day three post-caesarean section. We excluded women who received blood transfusion peri-operatively to allow donor-recipient haemoglobin equilibration to take place [28].

### Sample size estimation and sampling method

A sample size of 427 participants was estimated using the Kish Leslie formula for a single population proportion, n = z^2^ p (1-p) / d^2^ [29]. We considered a 95% confidence interval, a 5% margin of error, and a conservative 50% prevalence of severe anaemia post caesarean section (prevalence of severe anaemia after caesarean delivery is unknown in our setting). We factored a 10% non-response rate in the sample size calculation.

We used simple consecutive sampling to select the study participants.

### Data collection

Under the supervision of an Obstetrician, the trained midwife research assistants collected the data using a structured questionnaire that captured study-specific variables. Daily, after the morning ward round on the postnatal ward, research staff identified women on their third post-operative day, explained the study to them and invited them to participate. Women who passed the study eligibility criteria were taken through an informed consenting process before enrolment into the study.

### Laboratory procedures

To estimate haemoglobin concentration, about 5mls of blood from a superficial vein on the forearm was drawn into an EDTA vacutainer and taken to MRRH’s laboratory. The laboratory technologist measured the haemoglobin level using an automated meter (Sysmex XN-1000i® 5-part haematology analyser, *Sysmex America, Inc. Lincolnshire, Illinois, USA)* as described by Wang and colleagues [30]. For quality control, the analyser was cleaned and manufacturer-supplied controls run before testing samples. Using a closed mode of blood sampling, the analyser automatically sampled, processed, analysed blood and printed out haemoglobin level.

### Study variables

The outcome variable was severe anaemia defined as haemoglobin < 7.0 g/dl [10]. The independent variables were the woman’s demographic characteristics—age, marital status, occupation, residence, and level of education; medical characteristics—predelivery haemoglobin concentration (abstracted from woman’s chart), HIV serostatus, and history of malaria in the current pregnancy, diabetes; and obstetric characteristics—parity, gestational age, prenatal care attendance, iron supplement and duration, history of previous caesarean section, inter-delivery interval, history of pre-eclampsia, multifetal gestation. Other variables abstracted from the patient’s chart were: the type of caesarean section (emergency versus elective), indication for the caesarean section, birth weight (fetal macrosomia was defined as neonatal birthweight ≥4000 g [31]), and surgeon’s estimated blood loss.

For quality assurance, the questionnaire was pre-tested on pregnant women seeking antenatal care at the maternity ward of MRRH and the inconsistencies identified were corrected. The completed questionnaires were checked for completeness daily on-site and missing fields filled.

### Data entry and analysis

Completed questionnaires were entered into an EPI-Info (www.epidata.dk version 7.2.1) database and imported into STATA (StataCorp, College Station, Texas, U.S.A) version 15.0 for analysis. We described maternal baseline characteristics using means and standard deviation for continuous variables and proportions for categorical variables.

To determine the prevalence of severe anaemia post-caesarean section, haemoglobin concentration was categorized as severe anaemia (< 7 g/dl) and no severe anaemia (≥7 g/dl). We then calculated the proportion of women enrolled in the study who underwent caesarean section with haemoglobin concentration < 7 g/dl. To determine factors associated with severe anaemia, variables with *p*-value < 0.2 at bivariate logistic regression were entered into a multiple logistic regression model to determine factors independently associated with severe anaemia. A *p*-value less than 0.05 was considered statistically significant.

### Ethical consideration

Ethical approval was obtained from the Mbarara University Research Ethics Committee (MUST REC); Protocol reference number: 21/10–19. Written informed consent was obtained from all study participants. Adults aged ≥18 years and those aged < 18 years independently consented as emancipated minors in line with Ugandan guidelines [32]. The health workers in the postnatal ward were immediately informed about any woman who needed further evaluation and management of anaemia.

## Results

A total of 431 women were screened on day three post-caesarean section. We enrolled 427 participants and excluded 4 who received a blood transfusion in the immediate peripartum period. The mean age of the enrolled participants was 26.1 (±5.84) years). Ninety four percent (*n* = 401) were married, 73.3% (*n* = 313) attended at least four prenatal visits and 43.6% (*n* = 186) attained at least secondary level education. Other participants baseline characteristics are shown in Table 1.

**Table 1:** Baseline characteristics of women with/out severe anaemia after caesarean section at MRRH (*N* = 427)

Characteristics		Severe Anaemia		***P***-value
	Overall (N = 427)	Yes (***N*** = 29)	No (***N*** = 398)	
	n/N (%)	n/N (%)	n/N (%)	
Age in years, mean (±SD)	26.05(±5.84)	26.0(±7.52)	26.06 ± 5.70	0.955
Age category, n (%)				0.007
15–24	194(45.4)	16(55.2)	178 (44.7)	
25–34	188 (44.0)	6(20.7)	182(45.7)	
35+	45(10.5)	7(24.1)	38(9.6)	
Residence, n (%)				0.993
Rural	250(58.5)	17(58.6)	233(58.5)	
Urban	177(41.5)	12(41.4)	165(41.5)	
Marital status, n (%)				0.321
Single	26 (6.1)	3(10.3)	23(5.8)	
Married	401(93.9)	26(89.7)	375(94.2)	
Occupation, n (%)				0.634
Employed	104(24.4)	6(20.7)	98(24.6)	
Unemployed	323(75.6)	23(79.3)	300(75.4)	
Education, n (%)				0.164
No formal	43(10.1)	4(13.8)	39(9.8)	
Primary	198(46.4)	16(55.2)	182(45.7)	
≥ Secondary	186(43.6)	9(31.0)	177(44.5)	
ANC visits, n (%)				0.233
< 4	114(26.7)	5(17.2)	109(27.4)	
≥ 4	313(73.3)	24(82.8)	289(72.6)	
Parity, n (%)				0.261
Primiparous	190(44.5)	10(34.5)	180(45.2)	
Multiparous	237(55.5)	19(65.5)	218(54.8)	
Pre-caesarean Hb (g/dL), n (%)				< 0.001*
≥ 11	365(85.5)	14(48.3)	351(88.2)	
7.0–10.9	62(14.5)	15(51.7)	47(11.8)	
Prior caesarean sections, n (%)				0.001*
Index	228(53.4)	25(86.2)	203(51.0)	
Once	90(21.1)	1(3.5)	89(22.4)	
≥ 2	109(25.5)	3(10.3)	106(26.6)	
HIV status, n (%)				0.469
Negative	397(93.0)	26(89.7)	371(93.2)	
Positive	30(7.0)	3(10.3)	27(6.8)	
Caesarean section type, n (%)				0.053
Emergency	381(89.2)	29(100.0)	352(88.4)	
Elective	46(10.8)	0(0.0)	46(11.6)	
Delivered macrosomic foetus (%)				< 0.001*
No	401(93.9)	22(75.9)	379(95.2)	
Yes	26(6.1)	7(24.1)	19(4.8)	
Prior scar, n (%)				0.008*
No	287(67.2)	26(89.7)	261(65.6)	
Yes	140(32.8)	3(10.3)	137(34.4)	
Prolonged labour, n (%)				0.024*
No	324(75.9)	17(58.6)	307(77.1)	
Yes	103(24.1)	12(41.4)	91(22.9)	
Multiple pregnancy, n (%)				0.333
No	421(98.6)	28(96.6)	393(98.7)	
Yes	6 (1.4)	1(3.5)	5(1.3)	
Malpresentation, n (%)				0.198
No	382(89.5)	28(96.6)	354(88.9)	
Yes	45(10.5)	1(3.5)	44(11.1)	
Severe preeclampsia, n (%)				0.067
No	424 (99.3)	28(96.6)	396(99.5)	
Yes	3(0.7)	1(3.5)	2(0.5)	

*M* Mean, *SD* Standard deviation

Of the 247 enrolled participants, 29 had haemoglobin level < 7 g/dl, giving a prevalence of severe anaemia of 6.79 (95%CI: 4.78–9.61) per cent.

The factors independently associated with severe anaemia post caesarean section as presented in Table 2 were: having mild or moderate anaemia pre-caesarean section (aOR 9.6, 95%CI: 3.91–23.77, *p* < 0.01), and a macrosomic birth> 400 g (aOR: 7.9, 95% CI:2.18–28.85, *p* < 0.01).

**Table 2:** Crude and adjusted odds ratios of factors associated with severe anaemia after caesarean section at MRRH (N = 427)

Factor	Severe Anaemia		cOR(95%CI)	***P-value***	aOR(95%CI)	***P-value***
	Yes	No				
Age (years)						
15–24	16(55.2)	178(44.7)	Reference			
25–34	6(20.7)	182(45.7)	0.4(0.14–0.96)	0.041	0.6(0.19–1.70)	0.312
35+	7(24.1)	38(9.6)	2.0(0.79–5.32)	0.141	1.9(0.95–9.50)	0.149
Pre-caesarean section haemoglobin (g/dL)						
≥ 11.0	13(44.8)	352(88.4)	Reference			
7.0–10.9	16(55.1)	46(11.6)	9.4(4.26–20.81)	< 0.01	9.6(3.91–23.76)	< 0.01
Delivery of macrosomia foetus						
No	22(75.9)	379(95.2)	Reference			
Yes	7(24.1)	19(4.8)	6.3(2.41–17.70)	0.001	7.9(2.18–28.85)	0.002
Number of prior caesarean section						
None	24(82.8)	204(51.3)	Reference			
One	2(6.9)	89(22.3)	0.2(0.04–0.82)	0.026	0.3(0.06–1.67)	0.174
≥ 2	3(10.3)	105(26.4)	0.2(0.07–0.82)	0.023	0.7(0.14–3.57)	0.666
Indication for the current caesarean section						
Prior scar						
No	26(89.7)	258(64.8)	Reference			
Yes	3(10.3)	140(35.2)	0.2(0.06–0.72)	0.012	0.7(0.10–5.85)	0.174
Prolonged labour						
No	16(55.2)	308(77.4)	Reference			
Yes	13(44.8)	90(22.6)	2.8(1.29–6.00)	0.009	5.0(0.93–27.27)	0.060
Antenatal care visits						
< 4	5(17.2)	109(27.4)	Reference			
≥ 4	24(82.8)	289(72.6)	1.8(0.7–4.9)	0.239		
Parity						
Primiparous	10(34.5)	180(45.2)	Reference			
Multiparous	19(65.5)	218(54.8)	1.6(0.7–3.5)	0.264		
Preeclampsia						
No	28(96.6)	396(99.5)	Reference			
Yes	1(3.5)	2(0.5)	7.1(0.6–80.4)	0.255		
Malpresentation						
No	28(96.6)	354(88.9)	Reference			
Yes	1(3.5)	44(11.1)	0.3(0.04–2.2)	0.226		
Multiple pregnancy						
No	28(96.6)	393(98.7)	Reference			
Yes	1(3.5)	5 (1.3)	2.8(0.3–24.9)	0.354		
HIV						
Negative	26(89.7)	371(93.2)	Reference			
Positive	3(10.3)	27(6.8)	1.6(0.5–5.6)	0.472		

*OR* Odds Ratio, *aOR* Adjusted odds ratio, *CI* Confidence Interval, *p < 0.05

## Discussion

Our study showed that the prevalence of severe anaemia on the third-day post caesarean section at Mbarara Regional Referral Hospital was 6.79%. Given that most of the studies on anaemia are conducted during pregnancy, our study provides insights into the prevalence of severe anaemia after caesarean delivery in LICs’ settings where caesarean section rates are raising. In this study, the prevalence of severe anaemia after the caesarean section was higher than what was reported in Northern Uganda (0.8%), mid-western Uganda (0.5%) [33], as well as in postpartum women in North East Ethiopia (0.7%) [34] and India (2%) [35]. Also, our found prevalence is higher than reported one in 30 women in German who had severe anaemia (cut-off haemoglobin level < 8 g/dl) on the second day after delivery [17]. Moreover, a literature review found the prevalence of postpartum anaemia to range between 50 and 80% in developing countries [16]. The variations in prevalence of postpartum anaemia are due to disagreements among international clinical practice guidelines on cut-off diagnostic haemoglobin levels, the timing to test haemoglobin concentration, heterogeneity in populations of women at the individual level, plus the organisational setting-specific factors [8, 36]. Although an insignificant change in haemoglobin concentration among women who underwent noncomplicated caesarean section has been reported [37], it is important to determine haemoglobin level before hospital discharge in women after caesarean delivery.

In this study, mild or moderate anaemia pre-caesarean section and a macrosomic birth> 4000 g remained independently associated with severe anaemia post caesarean section at MRRH. Women with mild or moderate anaemia pre-caesarean section had up to ten times higher odds of suffering severe anaemia post-caesarean section—in agreement with other studies conducted at tertiary hospitals in sub-Saharan Africa [15, 38–40]. Similar findings were reported by a study conducted in Southwest Nigeria that showed up to twelve times higher odds of receiving blood transfusion among women with pre-caesarean anaemia [38]; and another observational study that found four times higher odds of suffering post-operative anaemia [39]. Also, a study in Egypt found haemoglobin below 11.0 g/dL to be a risk factor for primary postpartum haemorrhage up to seven times and subsequent severe anaemia postpartum regardless of the mode of delivery [40]. Low predelivery haemoglobin impairs transport of oxygen to the uterus, causes cellular dysfunction, a mechanism that can be used to explain impaired myometrial contractility, uterine atony and postpartum haemorrhage that aggravates pre-existing anaemia. Also, the preoperative anaemia that was present in the first place was aggravated by the blood loss during the caesarean section leading to post-operative anaemia.

In addition, this study found that pregnant women who had a macrosomic birth were eight times likely to develop severe anaemia after caesarean section. This was consistent with previous studies reporting an increased risk of postpartum haemorrhage and subsequent postpartum anaemia in women with macrosomia foetuses [40–42] including a study from Uganda. In the Ugandan study, the risk for postpartum haemorrhage was found to double after delivery of large babies over 4000 g, regardless of the mode of delivery [43], while in Egypt, a woman with macrosomia foetus was ten times likely to develop postpartum anaemia [42]. Foetal macrosomia leads to prolonged labour and other maternal complications, including operative delivery and postpartum haemorrhage, leading to subsequent severe anaemia. The increased physiologic vascularity of the pregnant uterus plus severed vessels during surgical access to the abdominal cavity confers a higher risk of postpartum haemorrhage and subsequent severe anaemia to women undergoing caesarean section.

Our study demonstrates that to diagnose severe anaemia earlier and institute proper treatment, women with a low preoperative Hb concentration and those whose foetus have macrosomia could be targeted for screening and haemoglobin optimisation before and during caesarean section.

Our study was not without limitations. We could not assess the association with estimated blood loss because surgeon-estimated blood loss was recorded in less than half of all participants. But, tendentially, the severe course of anaemia in these women was consistent with previous literature indicating a higher incidence of postpartum anaemia in women with excessive recorded or perceived intrapartum blood loss. Also, we excluded women who received blood transfusion which could have underestimated the prevalence of severe anaemia in this study. However, this was a small number and did not significantly alter our findings. Similarly, most studies on this subject matter rely on different thresholds of haemoglobin levels to classify anaemia. This makes it challenging to compare across studies. The findings of this study are only generalizable to women that undergo a caesarean section. Nonetheless, we demonstrate the prevalence of severe anaemia after caesarean section and provide insights into women at the highest risk for postoperative anaemia.

## Conclusion

In summary, we found that severe anaemia after caesarean section is uncommon in women undergoing cesarean section at our institution. It is associated with anaemia before surgery and delivery of a macrosomic foetus. In women delivered by caesarean section, we recommend haemoglobin determination before hospital discharge to diagnose and treat anaemia promptly.

## Supplementary Information

**Additional file 1.**

## Data Availability

De-identified data sufficient to produce primary study findings will be made available on reasonable request to the Department of Obstetrics and Gynecology, Mbarara University of Science and Technology. Data requests can be submitted through the corresponding author.

## Abbreviations

EDTA: Ethylenediamine tetra acetic acid
Hb: Haemoglobin
MRRH: Mbarara Regional Referral Hospital
MUST: Mbarara University of Science and Technology
REC: Research Ethics Committee
RBC: Red blood cells
UNCST: Uganda National Council for Science and Technology
